# Preliminary study of the technical limitations of automated breast ultrasound: from procedure to diagnosis

**DOI:** 10.1590/0100-3984.2019.0079

**Published:** 2020

**Authors:** Maria Julia Gregório Calas, Fernanda Philadelpho Arantes Pereira, Leticia Pereira Gonçalves, Flávia Paiva Proença Lobo Lopes

**Affiliations:** 1 Centro de Diagnóstico por Imagem (CDPI)/DASA, Rio de Janeiro, RJ, Brazil.; 2 CDPI Mulher - Centro de Diagnóstico por Imagem (CDPI)/DASA, Rio de Janeiro, RJ, Brazil.

**Keywords:** Ultrasonography, mammary/methods, Breast neoplasms/diagnostic imaging, Breast density, Early detection of cancer/methods, Mass screening/methods, Ultrassonografia mamária/métodos, Neoplasias da mama/diagnóstico por imagem, Densidade da mama, Detecção precoce de câncer/métodos, Programas de rastreamento/métodos

## Abstract

**Objective:**

To evaluate the main technical limitations of automated breast ultrasound and to determine the proportion of examinations excluded.

**Materials and Methods:**

We evaluated 440 automated breast ultrasound examinations performed, over a 12-month period, by technicians using an established protocol.

**Results:**

In five cases (1.1%), the examination was deemed unacceptable for diagnostic purposes, those examinations therefore being excluded.

**Conclusion:**

Automated breast ultrasound is expected to overcome some of the major limitations of conventional ultrasound in breast cancer screening. In Brazil, this new method can be accepted for inclusion in routine clinical practice only after its advantages have been validated in the national context.

## Abstract (pt)

**Objetivo:**

O objetivo deste estudo foi avaliar as principais limitações técnicas e a porcentagem de exames excluídos de ultrassonografia automatizada.

**Materiais e Métodos:**

Foram realizados 440 exames de ultrassonografia automatizada das mamas no período 12 meses, por técnicas, com protocolo estabelecido.

**Resultados:**

Em cinco casos (1,1%) a interpretação do estudo foi inaceitável, sendo o exame excluído do presente estudo para fins de diagnóstico.

**Conclusão:**

A ultrassonografia automatizada das mamas apresenta a expectativa de resolver importantes limitações da ultrassonografia convencional no rastreamento do câncer de mama, sendo necessária uma maior validação de dados brasileiros, para que este novo método seja aceito na prática clínica de rotina.

## INTRODUCTION

Mammography is still considered the best method for early detection of breast cancer. However, the complexity of evaluating breast structures using X-ray imaging methods and the subtlety of early lesions represent a challenge for specialists, particularly in cases of dense breasts^(1-6)^.

Although breast density is an independent risk factor for breast cancer, its “masking or obscuring” effect on mammograms hinders cancer detection^(1-8)^. In this context, an ultrasound examination of the breasts plays an important role as an adjunct to mammography and the clinical evaluation, and has been gaining ground as an important imaging test for detecting breast diseases. It is commonly performed as a complement to mammography in the screening of asymptomatic women with dense breasts, detecting additional early-stage or invasive cancer lesions^(1,3,9-12)^. However, like any other diagnostic method, ultrasound has problems related to its sensitivity and specificity. Its wider implementation is also curtailed by the fact that it is dependent on the skill of the operator and type of device employed. In addition, there is a shortage of trained ultrasound technicians and nonspecialist professionals often request ultrasound examinations for inappropriate indications^(6,9-12)^.

In Brazil, only physicians are trained and licensed to perform ultrasound, radiology technicians or technologists are currently not allowed to perform it. However, because of the high demand for breast ultrasound, ultrasound examinations of the breast are often performed by general radiologists or ultrasonographers with limited experience in breast imaging, rather than breast specialists, which reduces the sensitivity of the method and increases the number of false-positive results^(9-13)^. The growing concern of doctors and patients about the increased risk of breast cancer related to high breast density, combined with the limitations of mammography in dense breasts, has led to the development of additional screening tools, automated breast ultrasound (ABUS) being one such tool^(13-15)^.

The ABUS technique is a dedicated method that scans the breast in an automated, standardized manner with a transducer that is larger than that used in conventional ultrasound. Like mammography and magnetic resonance imaging, ABUS does not have to be performed by a physician^(13-15)^. In ABUS, image acquisition can be performed, in a standardized way with excellent resolution and an additional coronal plane, by radiology technicians, which allows physicians to spend their time interpreting the images and makes it possible to implement the method on a large scale. In 2012, ABUS was approved by the US Food and Drug Administration as a screening test for patients with dense breasts; however, it is not yet available at all imaging clinics in the United States^(16-20)^.

There are no published data regarding the performance of radiology technicians or technologists performing ABUS in Brazil. The primary objective of this study was to evaluate the main technical limitations of this method from the point of view of physicians and technicians. A secondary objective was to determine the proportion of examinations excluded from the analysis (rejected).

## MATERIALS AND METHODS

This was a prospective study in which we recruited consecutive women scheduled to undergo breast cancer screening (digital mammography and conventional ultrasound) at an imaging clinic. The following inclusion criteria were applied: having dense breasts, as determined by mammography and defined as Breast Imaging Reporting and Data System (BI-RADS) density category C or D^(2)^; and being asymptomatic. Women who were under clinical suspicion were excluded, as were those who had undergone surgery in the last 12 months. The study was approved by the local research ethics committee (Reference no. 1,728,661), and all participants gave written informed consent.

All of the women included in the study underwent digital mammography performed by a specialist radiology technician, with double reading by radiologists using the BI-RADS classification system, and conventional ultrasound performed and analyzed by either a specialist radiologist, a specialist ultrasound physician, or a general radiologist, also using the BI-RADS system.

**Figure 1:**
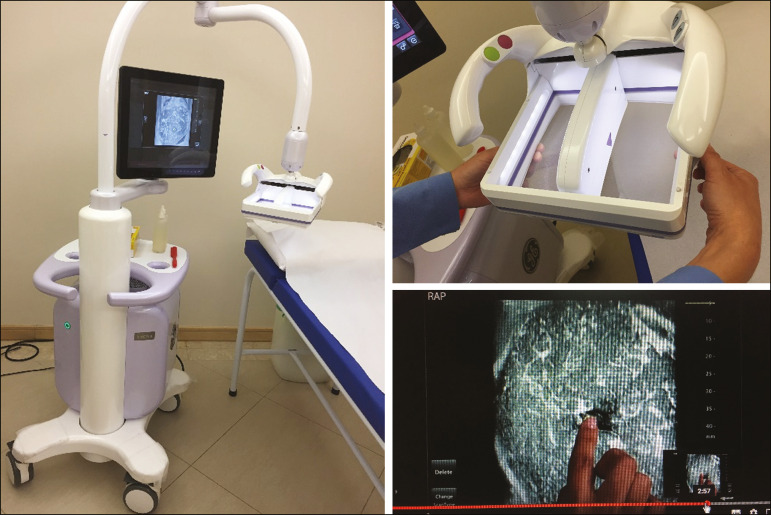
Images of the ABUS device used in the present study, showing the scanning transducer and screen on which the technicians check the views acquired and mark the nipple position.

All of the women evaluated underwent ABUS, performed by one of the radiology technicians, who followed a pre-established protocol. We employed the ABUS system depicted in Figure 1 (Invenia; GE Healthcare; Sunnyvale, CA, USA), which consists of a scanning unit and a diagnostic workstation. The scanning unit contains a high frequency (10-15 MHz) linear transducer.

For the examination, the patients were placed in the supine position with their arms extended above their head. The same gel used for conventional ultrasound was applied before the scan. The patient breast size was then selected from a standardized set of options (small, medium, or large) and light pressure was applied with the transducer. The breast tissue must be fully covered with gel to prevent air bubbles from forming on the contact surface.

**Figure 2:**
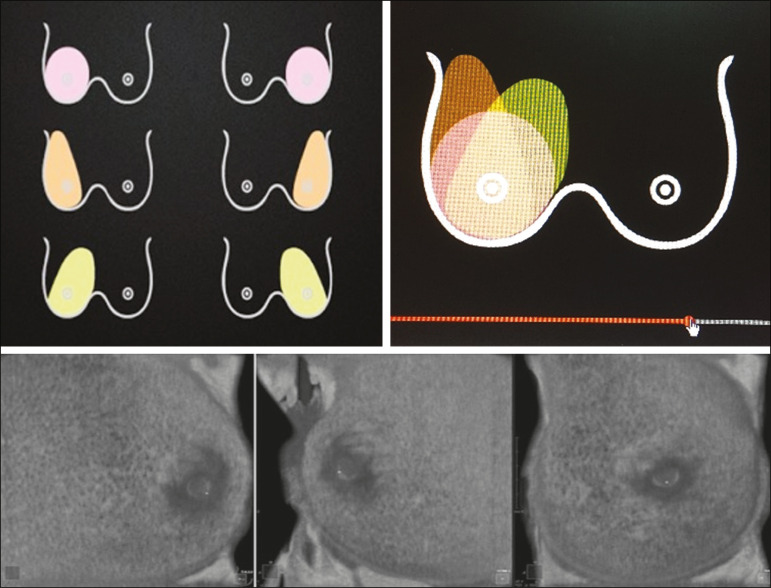
ABUS acquisitions (equivalent to mammography positionings or magnetic resonance sequences): lateral (orange), medial (yellow), and anteroposterior (pink).

The ABUS system sets up all ultrasound parameters automatically. The transducer slides seamlessly on a membrane, which is in constant contact with the breast. The number of scans required to cover the entire breast depends on its size. In our sample, it ranged from three to five scans per breast. As per the routine, anteroposterior, medial, and lateral views were obtained for all patients. In some cases, other views, such as superior and inferior views, were added. At the end of each scan, the operator marks the position of the nipple, which is used as a point of reference in all views, to enable correct orientation and post-processing reconstructions (Figures 1 and 2).

The images are acquired with a 15 cm field of view and an acquisition time of approximately 60 s per scan. After acquisition, the series of axial images are exported to a dedicated workstation and combined to form a three-dimensional ultrasound image that can be examined in multiplanar reconstructions (including coronal and sagittal images) of up to 2 mm in thickness, parallel to the chest wall. Other variables analyzed, but which were not objectives of this study, were the time the specialist technicians spent on bilateral scanning and the radiologist reading times.

All of the professionals involved (physicians and technicians) received technical, theoretical, and practical training standardized by GE Healthcare. Before the beginning of the study, each specialist technician received a minimum 30-day training encompassing a pre-established protocol, theoretical classes, and a practical component, in which they performed at least 20 examinations (not included in the study). The technicians stated that the following factors made it more difficult to perform the examination: a rigid breast, a large breast, a small breast, a flaccid breast, an elevated sternum, and complicated anatomy.

We selected a team of radiologists specializing in breast imaging to interpret the ABUS images. This medical team also received theoretical and practical training over a 30-day period, including weekly online tutorials. For image reading, they used the BI-RADS system and were blinded to the results of the other diagnostic methods^(1,2)^. The number of examinations was evenly distributed among the specialists (each analyzing approximately 80 cases). However, we did not perform an analysis of interobserver agreement among the ABUS findings.

The ABUS images were analyzed by our team of breast radiologists at workstations in appropriate image reading environments. We decided that an ABUS examination would be excluded if any portion of the breast was not well scanned or defined in such a way that it would jeopardize the complete visualization of the breast. The characteristic feature for exclusion was the presence of extensive acoustic shadowing from the superficial to the deeper planes, or the nonvisualization of a nodule or cyst in different planes (i.e., incomplete acquisition in any of the planes), which could result in a false-negative or false-positive result. The radiologists rejected the examinations independently of the findings of the mammogram and conventional ultrasound performed previously; patients were not negatively affected by that in any way. The specialist in breast radiology reported that the limiting factors for diagnosis included lack of compression, lack of coverage of a specific breast region, and artifacts; that is, all of the criteria that would lead to the nonvisualization of part of the breast and would have resulted in the examination being rejected because it would have been impossible for the physicians to write an appropriate report.

For the analysis of the main limitations of the method, the technicians filled out specific forms addressing the anatomical characteristics of the breasts, artifacts, and technical difficulties. The decision to exclude an examination and the final exclusion report were the sole responsibility of the radiologist who analyzed the case. We conducted a descriptive analysis of the technical limitations of all excluded examinations and calculated of the absolute frequency (proportion) of examinations excluded.

## RESULTS

We evaluated 440 ABUS examinations performed by the breast technicians between August 2017 and August 2018. Although all examinations were performed to full bilateral completion, technical difficulties were reported in 86 cases (19.5%) and difficulties in reading the images were reported in 30 cases (6.8%). All 30 of the examinations in which there were image reading difficulties also had technical limitations.

There were no significant differences among the radiology technicians who performed the ABUS in terms of the number of examinations excluded or in terms of the duration of the examination. The mean time for image acquisition was 14 min, ranging from 7 min and 30 s to 24 min (total examination time includes patient positioning and image acquisition). The mean radiologist reading time was 4 min and 25 s (range, 2-20 min).

From the point of view of the physicians, the factors limiting the use of ABUS (in 30 cases total) were lack of compression (in 21 cases), incomplete scanning of the breast (in 7 cases), and artifacts (in 2 cases). From the point of view of the radiology technicians, the limiting factors (in 86 cases) were a rigid breast (in 23 cases), a large breast (in 19 cases), a small breast (in 15 cases), a flaccid breast (in 14 cases), an elevated sternum (in 12 cases), and complicated anatomy (in three cases). Some of these difficulties were overcome, and the corresponding examinations were used in the study, with no impact on the diagnostic process.

**Figure 3:**
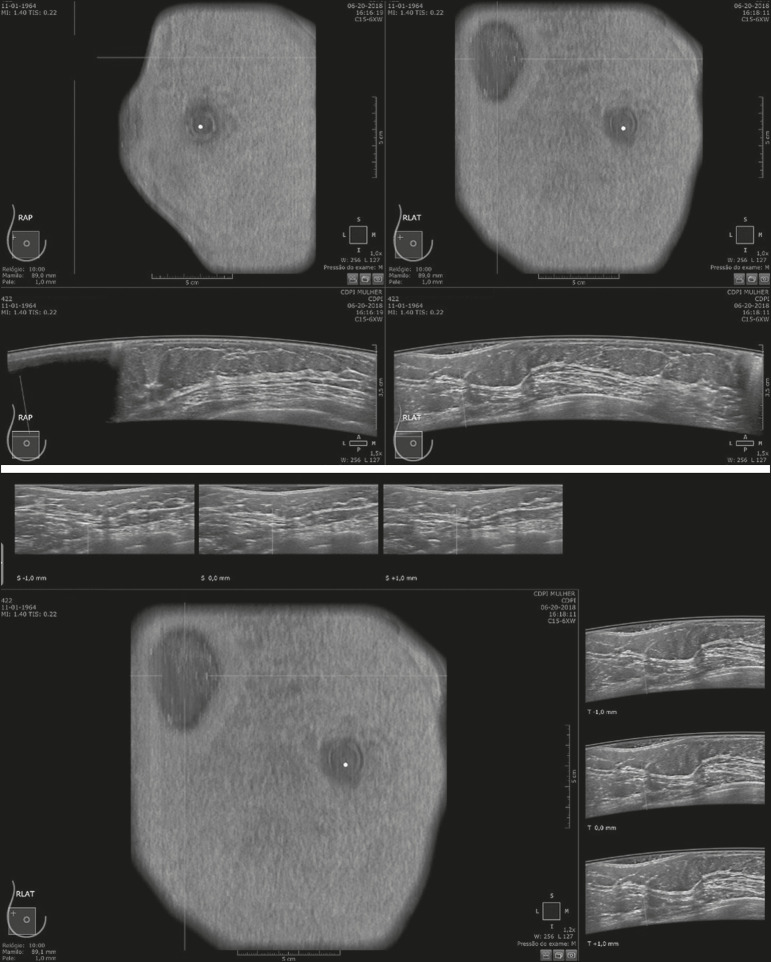
Acoustic shadowing artifact, caused by lack of proper contact, that can be easily visualized on the longitudinal axis. In the coronal plane, however, it appears as an easily identifiable pseudonodule.

**Figure 4:**
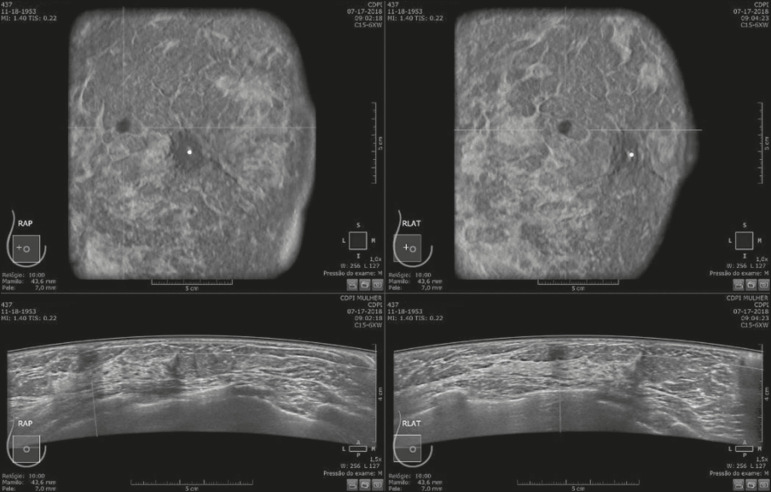
Insufficient gel. The image mimics a nodule or cyst but is actually an artifact.

**Figure 5:**
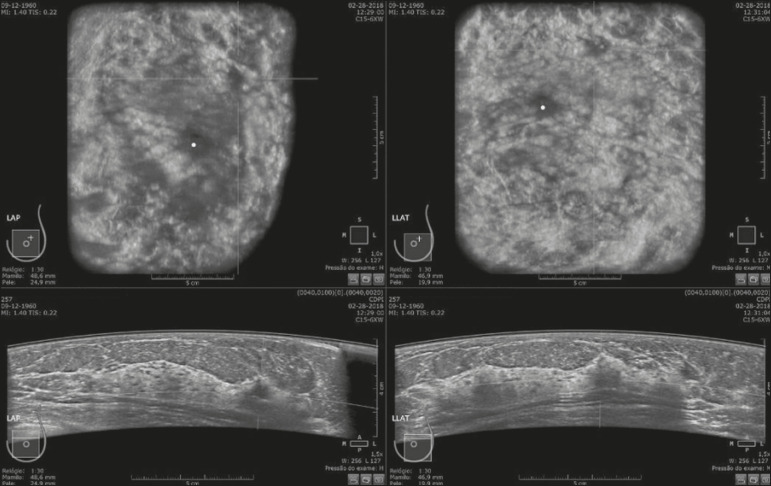
Artifact caused by inadequate compression, most commonly seen in dense breasts, which creates acoustic shadowing in deeper planes and can lead to suspicious findings.

Of the 440 cases, five were excluded because of technical limitations that would make it impossible to establish a diagnosis: four because of lack of breast compression (due to a rigid breast in three cases and to a large breast in one); and one because of lack of coverage of a region of interest (due to a small breast). Some of these cases are illustrated in Figures 3, 4, and 5. Despite the aforementioned technical difficulties, only five cases (1.1%) were deemed unacceptable for diagnostic purposes and were therefore excluded from the analysis.

## DISCUSSION

To perform a high-quality ultrasound examination, the operator must have full knowledge of the features of the device in use, mastery of the appropriate technique, experience with other imaging methods, and knowledge of the patient history. In breast radiology, observer agreement studies involving ultrasound are less numerous than are those involving mammography. Nevertheless, the interobserver agreement kappa indices described in the literature range from 0.28 to 0.83 for a diagnosis essentially based on the real-time subjective evaluation of the morphological aspects of a lesion^(10-12)^. Aiming to reduce the number of biopsies taken from benign solid tumors and increase the consistency of the diagnostic interpretation of ultrasound, several studies have proposed methods to aid in the diagnosis of breast cancer^(15,20-24)^.

As an auxiliary tool for breast cancer screening, ABUS has been the focus of several recent studies, complementing mammography in women with dense breasts. Automated ultrasound has the great advantage of being able to assess the entire breast in a standardized way, with the possibility of double reading. Therefore, it can be performed by radiology technicians, freeing physicians to spend more time reading images, on appropriate workstations. The ABUS images acquired by radiology technicians are automatically transferred to a dedicated workstation. They are then reconstructed and analyzed by a physician specializing in breast imaging. The images can be displayed in three dimensions (longitudinal, transversal, and coronal) or as a three-dimensional reconstruction, which is less useful for the diagnosis itself^(25-28)^.

In the present study, there were no predictable risks for patients, because automated ultrasound, like conventional ultrasound, is a noninvasive, low-risk method that does not expose patients to radiation. Digital mammography and conventional ultrasound examinations previously performed at the clinic at the request of the attending physician that were not directly related to this study did not interfere with the ABUS results. The ABUS examinations were performed as a courtesy, at no additional cost to patients. The examinations that were rejected for technical errors did not affect the evaluation of the patients, because their mammograms and conventional ultrasound were performed routinely, as per the physician requests. This study did not aim to evaluate the diagnostic accuracy of this new method, but rather to assess the real possibility of having it performed by a technician and later analyzed by a physician, as an ultrasound screening test in patients with dense or heterogeneously dense breasts.

In order to implement a large-scale ABUS screening program in which the examinations are performed by technicians, one must take into account one of the limitations of this new method, which is its inability to assess the axillary region; ABUS provides no information about the lymph node status^(29,30)^. Although this examination does not cover the axillary region, the main limiting factors described by the technicians in this study were lack of compression, lack of coverage of a breast region, and artifacts. Lack of axillary coverage was not a reason for exclusion of an examination in any of the cases.

The larger transducer employed in ABUS can hinder the contact of the scanning surface with the breast tissue and limit the evaluation of patients with firmer breasts, breast implants, or irregular breast contour because of previous surgeries and scarring, generating various artifacts, such as acoustic shadowing, that can hinder the visualization of peripheral lesions and lead to false-positive interpretations. However, ABUS should be recommended as a screening method for breasts found to be dense or heterogeneously dense on mammograms, and not for patients with implants or having recently undergone breast surgery. On the acquisition side, the main strategies for reducing artifacts include providing technicians with specific training on patient positioning and transducer contact; and instructing them to increase the pressure of the transducer and to change the angle of insonation. Once technicians or technologists are able to recognize acoustic shadowing caused by artifact, they can repeat the scanning immediately^(13,14,24,25)^.

Professionals will have a learning curve, and, over time, technicians will master the technique and generate fewer artifacts, whereas radiologists will find it easier to distinguish an artifact from a real nodule. Therefore, for improved diagnoses, the professionals who acquire and analyze the ABUS images must be familiar with the potential artifacts and be able to minimize them^(20-22)^.

It has been shown that properly trained radiology technicians are able to perform automated ultrasound^(23-28)^. If there is a need for diagnostic clarification, patients may be asked to return for additional image acquisition. In the present study, none of the patients whose ABUS examinations were excluded underwent additional examinations. However, that did not affect the patients, because they already had access to the results of the tests their physicians had requested (digital mammography and conventional breast ultrasound). As previously stated, only five (1.1%) of the examinations evaluated were excluded, corresponding to a 1.1% rate of patients who might need to return for additional image acquisition. That rate is within the acceptable limit and would not increase the psychological, financial, or institutional harm^(5,6,23)^.

There have been no studies attempting to determine whether ABUS is an operator-dependent test when performed by technicians in Brazil. Likewise, there have been no studies aimed at determining the prevalence of inconclusive reports due to errors in image acquisition. There are no such studies in the literature, because technicians in Europe and in the USA are licensed to perform ABUS and conventional ultrasound, also known as hand-held ultrasound^(16,24,25)^.

When conventional ultrasound is performed by physicians, the potential technical difficulties include the following: inadequate gain adjustment; inappropriate focal point; non-use of orthogonal images; inappropriate selection of transducer frequencies; lack of patient identification or incorrect patient identification; and inappropriate description of the location of a lesion. Acquiring the images with an insufficient amount of gel can also lead to errors, such as acoustic shadowing coming from the skin, which can hinder and even preclude the correct evaluation of a certain area on ultrasound. All of those potential difficulties are operator dependent; they hinder the diagnostic process and can lead to false-positive or -negative results^(20-22,25,28-30)^. Operator knowledge and experience depend on the number of tests the operator has performed throughout their professional life and on whether their scientific knowledge is kept up to date^(10-13,24)^. When ABUS is performed by technicians, parameters such as focus, depth, gain, use of harmonic imaging, and other features that improve spatial resolution are set automatically and there is therefore no need or indication for professionals to set them manually^(9,14,26-28)^.

The use of ABUS has enabled the American College of Radiology and the International Breast Ultrasound School to establish image quality and analysis criteria. Examples of automated ultrasound images were published in the latest American and Brazilian editions of the BI-RADS^(1-3,13,14)^.

## CONCLUSION

The use of ABUS as a screening tool is expected to overcome some important limitations of conventional ultrasound. With ABUS, the schedules of radiologists specializing in breast imaging can be optimized, whereas the dependence on operators and the variability of the results can be reduced. The possibility of requesting a second reading can also improve the quality of the diagnosis.

In the present study, the rate of examination rejection due to image acquisition errors was only 1.1%, which indicates that having technicians perform the examination is a real possibility, assuming that they have been properly trained. There is a need for further studies of this nature involving a greater number of technicians, patients, and centers, in order to validate our data and promote the acceptance of this new method into routine clinical practice in Brazil.
